# Role of the *ERas* gene in gastric cancer cells

**DOI:** 10.3892/or.2013.2417

**Published:** 2013-04-23

**Authors:** YANG LIU, ZHAOQIAN WANG, HUIMING LI, ZHAOPING WU, FANG WEI, HUIPING WANG

**Affiliations:** 1 Experimental Research Center, First People’s Hospital, School of Medicine, Shanghai Jiaotong University, Shanghai 200080, P.R. China; 2 Shanghai Gezhi High School, Shanghai 200001, P.R. China

**Keywords:** *ERas*, embryonic stem cell, gastric carcinoma, siRNA interference

## Abstract

As a novel member of the *Ras* family, *ERa*s, found in murine embryonic stem (ES) cells in 2003, was considered a pseudogene. To date, there are a few reports on the relationship between ERas and tumors. It was recently suggested that ERas could affect gastric carcinoma (GC) metastasis, but no significant relationship was found with tumor proliferation. Since *ERa*s plays an important role in tumor-like growth of ES cells subcutaneously injected into nude mice, we hypothesized that *ERa*s plays a role in tumor proliferation. In this experiment, we selected 7 GC strains from different sources with different differentiation degrees, we detected the expression of full length *ERas* transcript, and selected two *ERa*s highly expressing GC strains, MKN-28 and BGC-823. After knocking down the *ERa*s gene by siRNA, we observed that there was a significant decrease in proliferation, metastasis as well as clonality. Therefore, *ERa*s is confirmed to be an important gene in affecting tumor proliferation and metastasis. Furthermore, the significance of the *ERa*s mechanism and signaling pathway is shown.

## Introduction

The *ERa*s gene, which can support embryonic stem (ES) cell tumor-like growth (1–4), was first identified in murine ES cells by Takahashi *et al*(1). It encodes a protein of 227 amino acids with 43, 46 and 47% identity to conventional *Ras* oncogenes *H-ra*s, *K-ra*s and *N-ra*s, respectively, defined as a new member of the *Ras* family. Unlike other *Ras* family members, the *ERa*s product is a constitutively active protein without any mutation (1), while conventional *ras* oncogenes acquire activation of carcinogenesis by point mutation of several amino acids, which include Gly12, Ala59 or Glu63 (5,6). The ERas protein contains amino acid residues identical to those present in active mutants of the conventional *Ras* oncogenes *K-ra*s, *N-ra*s and *H-ra*s (7). The conventional *Ras* members mainly function through activating either phosphatidylinositol-3-OH kinase (PI3K) or Raf pathway (8,9), while ERas interacts with PI3K (10) but not with Raf (11,12). The human *ERas* gene was initially erroneously recognized as a processed pseudogene *HRa*sp (*Ha-Ras*2) (13,14) with deficiency or meaningless mutation, but it was more recently described as a gene potentially encoding a functional human ERas protein (15–18). Therefore, a few studies on human *ERa*s have previously been reported.

In 2002, Bjorklund *et al*(19) found that mouse ES cells developed into teratomas when transplanted into nude mice. In 2003, Takahashi *et al*(1) found that *ERa*s is key in the tumor-like growth properties of ES cells. In 2009, Kaizaki *et al*(17) discovered *ERa*s was actively expressed in gastric cancer (GC) and was closely related to its oncogenesis. These results indicate that the expression of *ERas* may be associated with cell proliferation and transformation.

However, in 2010, Kubota *et al*(20) detected 142 clinical samples of GC, which showed that *ERa*s expression was strongly associated with liver and lymph node metastases. Meanwhile, there was no significant correlation between *ERa*s expression and histological differentiation. Overexpression of *ERa*s in GC cell lines promoted colony formation while it showed no significant effect on cell proliferation. These results indicated that *ERa*s was only associated with the metastasis of GC. However, clonality is an indicator of proliferation capacity. Hence, considering the multiaspect influences of oncogenesis, we decided to knock down the *ERa*s gene in order to study the impactions on proliferation and clonality of GC cells.

In addition, Kameda and Thomson (2) used RT-PCR to analyze expression of the *ERa*s gene in human ES cells in 2005, and they could not detect a full-length *ERa*s coding transcript. Instead, a truncated noncoding transcript was found, which was caused by a premature polyadenylation signal predicted through sequence analysis and confirmed by 3′RACE analysis. Except for the premature PloyA locus, humans and chimpanzees have typical-Alu-s-retrotransposon insertions, which also influence the expression of *ERa*s at this specific locus. Moreover, the lack of *ERa*s expression in human ES cells indicates that the oncogenesis is very different from that of murines (21). These findings indicate that further studies should be performed to ascertain whether or not a full-length *ERas* coding transcript is present in human GC cells.

In this study, 8 cell strains from different sources, GC lymph node metastasis, GC liver metastasis, GC ascites and GC tissues, with different differentiation degrees, were chosen to determine whether a full-length *ERa*s mRNA exists and to elucidate the difference of degree of *ERa*s expression among these GC strains by RT-PCR, real-time PCR and western blotting. Furthermore, we confirmed the effect of *ERas* knockdown on cell proliferation, metastasis and clonality in *ERas* highly expressed GC strains.

## Materials and methods

### Cell culture and cell lines

The cell lines GES-1, MKN-28, MKN-45, BGC-823, NCL-N87, SNU-16, SGC-7901 and AGS were cultured in RPMI-1640, supplemented with 10% fetal bovine serum (FBS; HyClone, Logan, UT, USA). They were cultured in an atmosphere of 5% CO_2_ at 37°C. BGC-823, NCL-N87 and AGS cells were obtained from Shanghai Institute of Cell Bank (Shanghai, China); GES-1, MKN-28, MKN-45, SNU-16 and SGC-7901 were purchased from the American Type Culture Collection (ATCC, Rockville, MD, USA).

### Amplification of the ERas full-length transcripts and sequencing analysis

Total cellular RNA was extracted from each cell line with TRIzol^®^ (Invitrogen Life Technologies, Carlsbad, CA, USA), and cDNA was synthesized with Reverse Transcription kit (Promega Corp., Madison, WI, USA), and both were performed according to the manufacturer’s protocol. The cDNA was synthesized by PCR with PrimeSTAR HS DNA polymerase (Takara Bio, Shiga, Japan). The primers used for the full-length *ERas* coding sequence were: forward, 5′-atggagctgccaacaaagcctggca-3′ and reverse, 5′-ttcaggccacagagcagccacagt-3′, which gave an amplified fragment of 702 bp. The reaction conditions were: 94°C for 15 sec, 62°C for 45 sec and 72°C for 30 sec, repeated for 35 cycles. The products were separated by 1.2% agarose gel electrophoresis, and the 702 bp bands were recycled using aqua-Spin gel extraction mini kit (Watson Biotechnologies, Inc., Shanghai, China). The identification of PCR products was confirmed by sequencing analysis (Songon Biotech Co., Shanghai, China).

### Real-time quantitative PCR

The real-time quantitative PCR analyses were performed in triplicate using SYBR^®^ Premix Ex Taq™II kit (Takara Bio). The *GAPDH* gene was chosen as an endogenous control. The primers used for *ERas* were: forward, 5′-cacatggagcccttccttc-3′ and reverse, 5′-tgtccagggtcaactccttc-3′; the primers used for *GAPDH* were: forward, 5′-ggacctgacctgccgtctag-3′ and reverse, 5′-gtagcccaggatgcccttga-3′; the PCR conditions were as follows: 94°C for 15 sec, 58°C for 45 sec, 72°C for 20 sec, repeated for 35 cycles. Amplified products were separated by 0.9% agarose gel electrophoresis.

### Western blot analysis

Cells were lysed in a lysis buffer containing 2% sodium dodecyl sulfate (SDS) and 0.125 M Tris-HCl (pH 6.8) on ice for 30 min, followed by high-speed centrifugation, and the supernatant protein was finally collected. SDS-PAGE was performed using 10% polyacrylamide gels. PAGE separated proteins were electrophoretically transferred onto nitrocellulose membranes. The membrane filters were blocked with 5% powdered milk in TBST (0.1% Tween-20) for 2 h and then incubated in rabbit ERas antibody (Abgent, Suzhou, China) diluted 1:100 in TBST at 4°C overnight, and finally incubated with HRP anti-rabbit secondary antibody (Kangchen Biotech, Shanghai, China) diluted 1:2,000 for 1 h at room temperature. Antigens on the membrane were detected with enhanced chemiluminescense detection reagents (Roche, Basel, Switzerland).

### Small interfering RNA transfection

Two *ERas* stealth siRNA, no. 30 forward, GCAACUAGCUUUGAGGGAC(dTdT) and reverse, GUCCCUCAAAGCUAGUUGC(dTdT); no. 32 forward, GUAACAUGGGAGUGCCUAA(dTdT) and reverse, UUAGGCACUCCCAUGUUAC(dTdT); one high GC% negative control siRNA forward, CCUACGCCACCAAUUUC GU(dTdT) and reverse, ACGAAAUUGGUGGCGUAGG (dTdT) were designed and synthesized (Bioneer, Daejon, Korea). siRNA was mixed with Lipofectamine™ 2000 (Invitrogen Life Technologies) in an OptiMEM serum-free medium (HyClone) for 30 min at room temperature and then added to each 24-well plate containing MKN-28 or BGC-823 cells. Cells were maintained in a humidified 5% CO_2_ incubator at 37°C for 6 h with the old medium being replaced by a fresh medium. After 24 h of transfection, cells were harvested for cell proliferation, migration and colony formation assays.

### CCK-8 assay

siRNA-transfected MKN-28 and BGC-823 cells were seeded into 96-well plates at a density of 3×10^3^ cells/well and maintained in culture medium for 5 days. Each well set five duplicates. We measured cell growth using cholecystokinin (CCK) assay by Cell Counting Kit (Dojindo, Tokyo, Japan) according to the manufacturer’s instructions.

### Cell migration assay

For wound-healing experiments, MKN-28 and BGC-823 cells transfected with siRNA were cultured to 80% confluence after being seeded into 6-well plates, then scraped using a p10 tip (time 0), and suspended cells were washed with PBS three times. Cells were incubated for another 4 days and images were captured by microscope (Zeiss, Oberkochen, German) at the same time every day. Migration distance was measured from images (5 fields) at each indicated time point.

Transwell assay of MKN-28 and BGC-823 cells was assessed using 6.5 mm diameter inserts (Corning Costar Corp., Corning, NY, USA). A total of 3×10^4^ cells were suspended in 100 μl serum-free RPMI-1640 medium and loaded into upper wells; lower chambers were filled with 600 μl of complete medium (RPMI-1640 supplemented with 10% FBS). Migration chambers were incubated in a humidified 5% CO_2_ incubator at 37°C for 24 and 48 h. Cells were then fixed with 600 μl of paraformaldehyde for 20 min. The inner surfaces of the upper chambers were wiped using cotton swabs to remove non-migrated cells in the migration assay. The chambers were then washed with PBS and stained with 500 μl crystal violet for 20 min at room temperature. Stained cells were counted using the ImageJ software, and 5 random fields were counted (Zeiss).

### Colony formation assay

A total of 500 siRNA-transfected MKN-28 and BGC-823 cells were seeded in 6-well plates and incubated for 14 days respectively, with the medium replaced every 4 days. On the 15th day, the cells were stained with crystal violet for 20 min and washed with tap water for 10 min. For each dish, colonies in five random fields were counted using the ImageJ software.

### Statistical analysis

Each measurement was performed in triplicate. Original real-time PCR data, CCK-8 data, migration/invasion data and colony formation data were recorded as continuous variables and analyzed using Student’s t-test or linear polynomial ANOVA with LSD post hoc examination. All statistical analyses were performed using SPSS 16.0 software. P-values <0.05 were considered to indicate statistically significant differences.

## Results

### ERas expressed in GC cells

**Figure 1:**
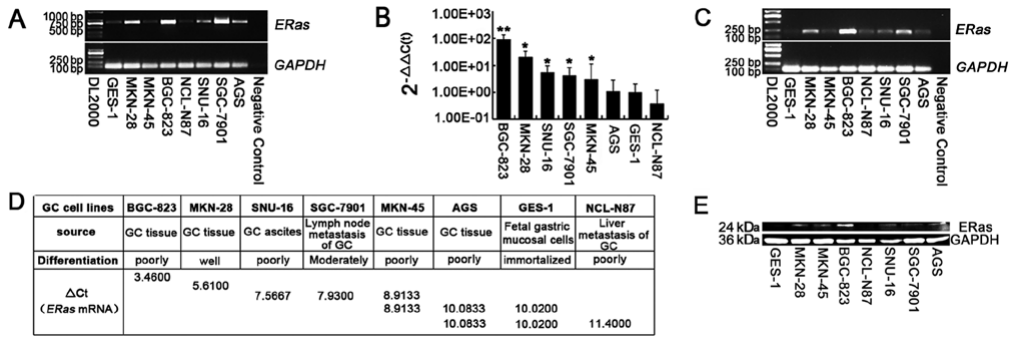
*ERas* mRNA and protein expression in seven gastric cancer cell lines and the GES-1 gastric mucosa cell line. (A) The full-length *ERas* transcript was examined by RT- PCR. The negative control indicates no template in the reaction. (B) The quantitative expression of the *ERas* gene was examined by real-time PCR. (C) The electrophoretogram of the real-time PCR product. (D) The source and differentiation of gastric cancer cell lines and its ΔCt value of *ERas* mRNA. All eight cell lines were divided into five groups by one-way ANOVA according to the degree of *ERas* mRNA expression. (E) Expression of ERas protein in these cell lines was determined by western blotting. The *GAPDH* gene was used as an internal control (^*^P<0.05, ^**^P<0.01 vs. GES-1 cells).

The full-length *ERas* mRNA transcript was detected in all seven GC cell lines and GES-1 gastric mucosa cell line by RT-PCR. The forward and reverse primers were located in open reading frame (ORF) 1–25 and 680–702 bp separately, which give rise to an amplified fragment of 702 bp (Fig. 1A). To assess whether *ERas* expression was different with mutant *H-ras*, *K-ras* and *N-ras*, mutation analysis was performed by sequencing analysis, revealing no mutation of *ERas* in all the gastric cell lines that we selected (data not shown).

The expression levels of *ERas* mRNA were also determined by real-time PCR. All eight cell lines were divided into five groups by one-way ANOVA. The GC cell line BGC-823 expressed most highly (P<0.01), MKN-28 expressed highly (P<0.05), SNU-16, SGC-7901, MKN-45 expressed moderately (P<0.05), while AGS, NCL-N87 expressed poorly, similar to the normal gastric mucosal cell line, GES-1 (P>0.05) (Fig. 1B-D). ERas protein expression was also confirmed by western blotting (Fig. 1E).

### ERas increases GC cell proliferation

To examine the role of ERas in cell proliferation, we measured BGC-823, MKN-28 cell growth by CCK-8 assay after transfecting with *ERas* siRNA30 and siRNA32.

**Figure 2:**
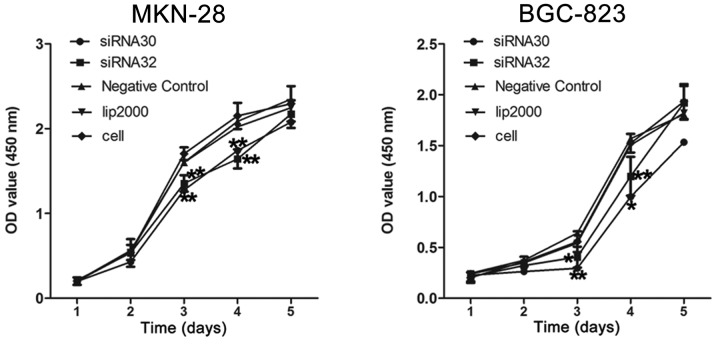
The role of *ERas* on gastric cancer cell line proliferation. MKN-28 and BGC-823 cells treated with *ERas* siRNA 30, *ERas* siRNA 32, negative control siRNA, lipofectamine 2000 only and cell only respectively were plated at 3×10^3^ cells/well on 96-well plates. OD value (450 nm) was measured by CCK-8 assay at 4, 24, 48, 72 and 96 h, shown as mean ± SD (^*^P<0.05 and ^**^P<0.01 vs. control).

We observed a significant decrease in cell proliferation when the expression of *ERas* was knocked down in MKN-28 and BGC-823 cells (Fig. 2).

On the third day, the OD value of MKN-28 cells decreased from 1.60±0.05 to 1.28±0.10 when transfected with siRNA30 (P<0.01), and it decreased to 1.35±0.10 when transfected with siRNA32 (P<0.01). Meanwhile, the OD value of BGC-823 cells decreased from 0.64±0.18 to 0.30±0.08 and 0.40±0.10 when transfected with siRNA30 (P<0.01) and siRNA32 (P<0.05) individually.

On the fourth day, the OD value decreased from 2.09±0.09 to 1.74±0.10 and 1.64±0.11 individually in MKN-28 cells when transfected with siRNA30 (P<0.01) and siRNA32 (P<0.01), respectively, while it decreased from 1.57±0.07 to 1.00±0.46 (P<0.05) and 1.20±0.19 (P<0.01) in BGC-823 cells. These data indicate that the proliferation of these GC cell lines is significantly promoted by the *ERas* gene.

### ERas promotes GC cell migration

We also confirmed the effect of ERas on migration in MKN-28 and BGC-823 cells by Transwell and wound-healing assay.

**Figure 3:**
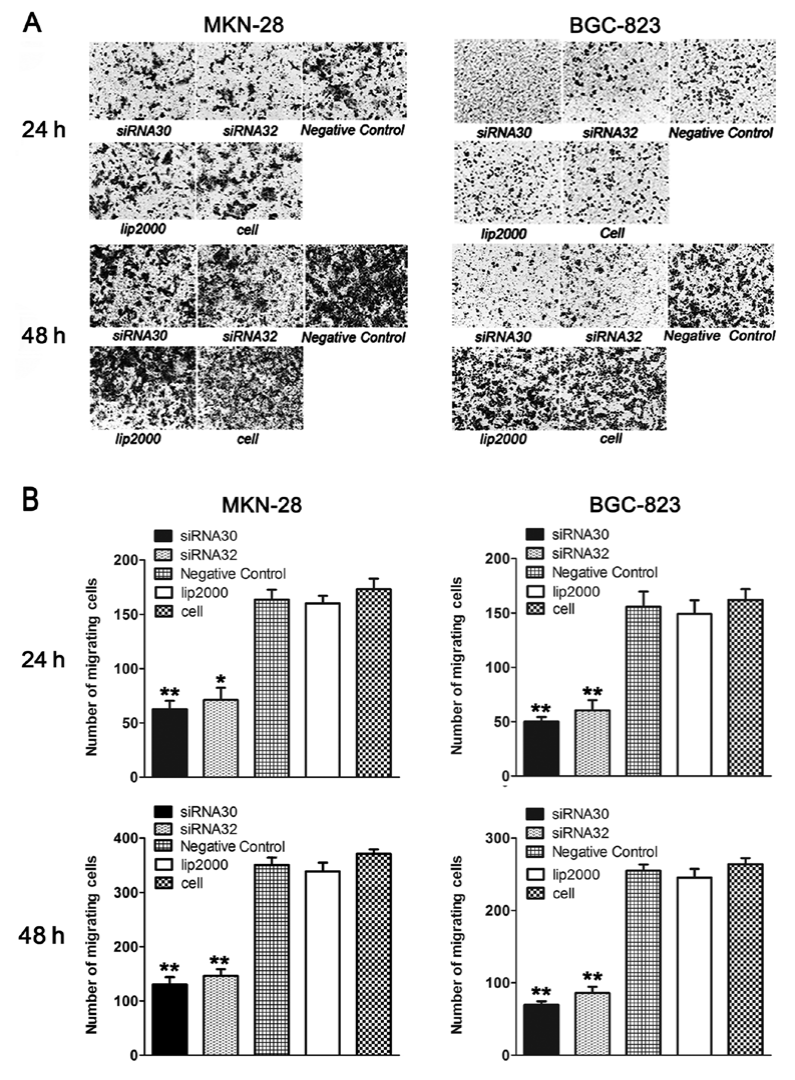
The role of *ERas* in MKN-28 and BGC-823 cell migration by Transwell assay. After 24 h of siRNA30, siRNA32 and negative control transfection, 3×10^4^ cells were transferred into 6.5 mm inserts and incubated for another 24 and 48 h. (A) Cells were stained with crystal violet and observed by microscope (x50 magnification; Zeiss). (B) The number of migration cells in five random fields was counted using the ImageJ software (x100 magnification; Zeiss) and shown as mean ± SD (^*^P<0.05 and ^**^P<0.01 vs. control).

As shown in Fig. 3A and B, knockdown of *ERas* by siRNA significantly impaired the ability of MKN-28 and BGC-823 cells to migrate through the membranes. Twenty-four hours later, the number of migratory cells decreased from 163.5±9.19 to 62.50±7.78 (P<0.05) and 71±11.31 (P<0.01) when transfected with siRNA30 and siRNA32 in MKN-28 cells, while it decreased from 155.50±9.19 to 50±4.24 (P<0.01) and 60.5±9.19 (P<0.05) in BGC-823 cells. Forty-eight hours later, the number of migratory MKN-28 cells decreased from 350.5±13.44 to 62.50±7.78 (P<0.01) and 71±11.31 (P<0.01) respectively, while the number of migratory BGC-823 cells decreased from 255±8.49 to 69.5±4.95 (P<0.01) and 86±8.49 (P<0.01).

**Figure 4:**
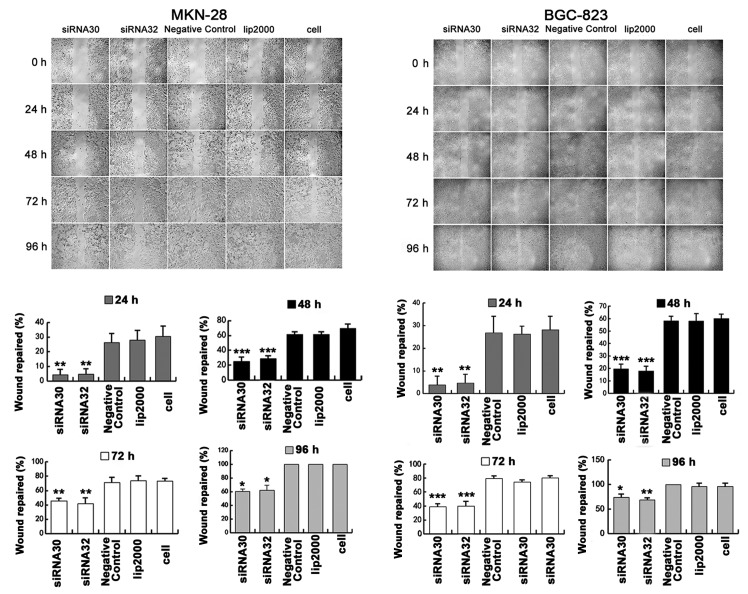
The role of *ERas* in wound healing ratio in MKN-28 and BGC-823 cells. After 24 h of transfection with *ERas* siRNA30, siRNA32 and negative control, cells were scraped with p10 tip (time 0) and images were captured every day at the same time point (x50 magnification; Zeiss). Migration distance was measured from images (5 fields) captured at each indicated time point. Wound repair percentage of each cell line is shown using bar charts (^*^P<0.05, ^**^P<0.01 and ^***^P<0.001 vs. control).

Then, we further analyzed migration ability by using wound-healing for 4 days. As shown in Fig. 4, the speed of wound repair was markedly slower when *ERas* was silenced by siRNA in MKN-28 and BGC-823 cells at 24, 48, 72 and 96 h. The statistical significance was most notable at 48 h, when the wound repair percentage decreased from 61.35±3.54% to 25±6.25% (P<0.001) and 28.89±3.85% (P<0.001) after *ERas* was knocked down by siRNA30 and siRNA32 respectively in MKN-28 cells, while the percentage decreased from 58.13±3.61% to 19.61±3.78% (P<0.001) and 18±3.85% (P<0.001) respectively in BGC-823 cells.

### ERas promotes GC cell colony formation

**Figure 5:**
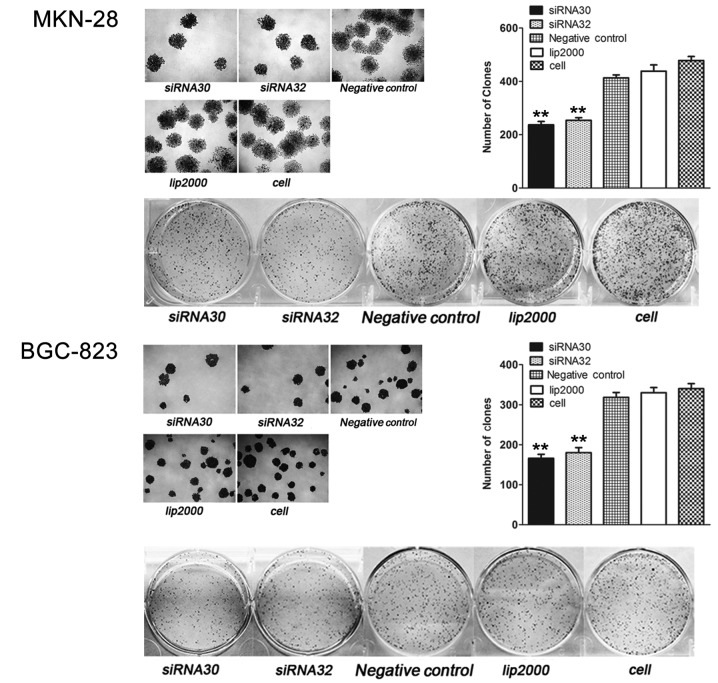
The effect of ERas on colony formation ability. After 24 h of siRNA30, siRNA32, negative control transfection, 5×10^2^ cells were transferred into 6-well plates and incubated for 14 days. The cells were stained with crystal violet, photographed by a microscope (x50 magnification; Zeiss) and an ordinary camera. Bar charts show the number of colonies; significances were analyzed by using the Student’s t-test (^**^P<0.01 vs. control).

The colony formation was assessed two weeks later. As shown in Fig. 5, *ERas* knockdown by siRNA30 or siRNA32 reduced the number of colonies from 413±11.31 to 237±12.73 (P<0.01) and 254±9.90 (P<0.01) in MKN-28 cells, while the number of colonies in BGC-823 cells was reduced from 318.5±12.02 to 166±9.90 (P<0.01) and 180±12.73 (P<0.01).

## Discussion

*The ERas* gene is strongly expressed in BGC-823 and MKN-28 cell strains among the eight gastric cell lines (Fig. 1B-D). To examine the effect of ERas on GC cell proliferation, these two highly expressing endogenous *ERas* cell strains were investigated after treatment with *ERas* siRNA by CCK-8 assay for 5 days. Knockdown of *ERas* inhibited the proliferation ability markedly compared to control in BGC-823 and MKN-28 cells on the third and fourth day (Fig. 2). Kubota *et al*(20) concluded that ERas could not promote the proliferation in GCIY cells transfected with an *ERas*-overexpressing vector by MTS assays for 6 days, whereas it could enhance colony formation as the cell clonality experiment results showed. They came to the same result that there was no relationship between proliferation and the *ERas* gene in GCIY and NUGC-4 cells with *ERas* knocked down by stealth siRNA using MTS assays for 2 days. However, whether or not knockdown of *ERas* inhibits cell colony formation was not further established. GCIY cell strain was used in both *ERas* overexpression and knockdown experiment. However, the expression level of *ERas* in this strain was relatively low, indicating it was not suitable for the knockdown experiment. On the other hand, the observation period in their study was relatively short. These reasons may lead to the disparity. Meanwhile, the results of Transwell test, scratch test and colony formation test (22,23) proved that ERas is able to increase GC cell migration and colony formation which were also two aspects that could reflect cell proliferation. Hence, there is sufficient evidence to prove that ERas enhances GC cell proliferation.

Furthermore, *ERas* was expressed most highly in poorly differentiated BGC-823 cells and well differentiated MKN-28 cells, less highly in poorly differentiated SNU-16 cells, moderately differentiated SGC-7901 cells and poorly differentiated MKN-45 cells, almost silently in poorly differentiated AGC cells, immortal GES-1 cells and poorly differentiated NCL-N87 cells (Fig. 1A-C). From these date, the conclusion that expression of *ERas* is not related to histological differentiation is the same as that of Kubota *et al*. However, from analyzing the following seven cell strains, SGC-7901 from GC lymph metastasis, NCL-N87 from GC liver metastasis, SNU-16 from GC ascites, GES-1 from fetal gastric mucosa and others from GC tissues, the connection between ERas and metastasis of gastric lymph and liver is not so strong, which is different from the result of Kubota *et al*. Therefore, to fully understand the connection between ERas and gastric lymph metastasis and liver metastasis, further research is required.

The full-length transcript of *ERas (*2,20) in those 7 GC cell strains were examined (Fig. 1A), suggesting that activated ERas is present universally in GC cells. Since a full-length transcript cannot be found in human ES cells, there should be some common activation factors which inhibit the prematuration of early polyadenylation signal and the insertion of Alu-S transposons to activate *ERas* expression.

To date, there are few studies on the relationship between ERas and cancer, and the function of ERas has yet to be determined. This study used CCK-8, Transwell, scratch test and clonality test to successfully prove that ERas has the ability to enhance GC cell proliferation, metastasis and clonality. Compared to Kubota *et al*(20), this study proved that the activation of the *ERas* gene in GC cells is common and the function of ERas in the processes of GC cell development and metastasis is important; there must be a significance of clarifying the correlative key signal pathways and the cytokines to activate the pathways.

## Abbreviations

ES: embryonic stem
PI3K: phosphatidylinositol-3-OH kinase
GC: gastric carcinoma
